# Constructive Neuroengineering of Axon Polarization Control Using Modifiable Agarose Gel Platforms for Neuronal Circuit Construction

**DOI:** 10.3390/gels11080668

**Published:** 2025-08-21

**Authors:** Soya Hagiwara, Kazuhiro Tsuneishi, Naoya Takada, Kenji Yasuda

**Affiliations:** 1 Department of Pure and Applied Physics, Graduate School of Advanced Science and Engineering, Waseda University, 3-4-1 Okubo, Shinjuku, Tokyo 169-8555, Japan; soyahagiwara@ruri.waseda.jp (S.H.); shikon.purple@fuji.waseda.jp (N.T.); 2 Department of Physics, School of Advanced Science and Engineering, Waseda University, 3-4-1 Okubo, Shinjuku, Tokyo 169-8555, Japan; kazu140613@akane.waseda.jp

**Keywords:** neuron, hippocampal neuron, axon polarization, neurite elongation control, agarose gel micropattern, constructive neuroengineering

## Abstract

Axon polarization is a fundamental process in neuronal development, providing the structural basis for directional signaling in neural circuits. Precise control of axon specification is, thus, essential for the bottom-up construction of neuronal networks with defined architecture and connectivity. Although neurite length and elongation timing have both been implicated as determinants of axonal fate, their relative contributions have remained unresolved due to technical limitations in manipulating these factors independently in conventional culture systems. Here, we developed a constructive neuroengineering platform based on modifiable agarose gel microstructures that enables dynamic, in situ control of neurite outgrowth length and timing during neuronal cultivation. This approach allowed us to directly address whether axon polarization depends primarily on neurite length or the order of neurite extension. Using a single-neurite elongation paradigm, we quantitatively defined two length thresholds for axon specification: a critical length of 43.3 μm, corresponding to a 50% probability of axonal differentiation, and a definitive length of 95.4 μm, beyond which axonal fate was reliably established. In experiments involving simultaneous or sequential elongation of two neurites, we observed that neurite length—not elongation order—consistently predicted axonal identity, even when a second neurite was introduced after the first had already begun to grow. The presence of a competing neurite modestly elevated the effective critical length, suggesting inhibitory interactions that modulate length thresholds. These findings provide the first direct experimental confirmation that neurite length is the primary determinant of axon polarization and demonstrate the utility of constructive microfabrication approaches for dissecting fundamental principles of neuronal polarity. Our platform establishes a powerful experimental foundation for future efforts to achieve complete control over axon and dendrite orientation during the engineered construction of functional neuronal circuits.

## 1. Introduction

The establishment of neuronal polarity, in which neurons differentiate structurally and functionally distinct axons and dendrites, is a fundamental process for the formation of proper neural circuits. Among these processes, axon polarization, the specification of a single neurite as the axon, represents a critical early event that determines the directionality of neuronal signaling and connectivity [1,2].

Pioneering studies using cultured hippocampal neurons revealed that initially equivalent minor neurites undergo asymmetric growth, with one becoming the axon through a coordinated sequence of cytoskeletal remodeling and molecular signaling events [3,4]. Microtubule dynamics play a central role in this polarization process. In particular, the selective stabilization of microtubules within a nascent neurite is a key determinant of axon specification [5,6,7], highlighting the importance of cytoskeletal regulation at early stages.

In parallel, intracellular polarity proteins such as PAR-3, PAR-6, and atypical protein kinase C (aPKC) have been shown to accumulate at the tip of the future axon, orchestrating downstream signaling cascades that define axon [8,9,10,11]. Small GTPases including Cdc42 and Rac1 further contribute to the establishment of neuronal polarity by regulating actin dynamics and directing vesicle trafficking [4,12,13]. Emerging evidence also implicates centrosome positioning and non-centrosomal microtubule organization as factors that bias axon formation [14,15,16,17]. Moreover, the axon initial segment (AIS) serves as a critical compartment for maintaining axonal identity by controlling selective membrane protein distribution and endocytosis [18,19], underscoring the dynamic interplay between membrane trafficking and cytoskeletal organization.

An important question that has emerged concerns whether axon polarization is determined by the length of a neurite or by the order in which neurites extend. Experimental studies have demonstrated that axon specification occurs preferentially in the longest neurite at the time of polarization, regardless of the sequence of neurite outgrowth [1,3]. This suggests that it is the relative length, rather than the temporal order of extension, that serves as a critical cue for axon selection. The longest neurite may provide a favorable structural and molecular environment—such as enhanced microtubule stabilization or polarized membrane trafficking—that biases it toward axonal identity. In cultured rat hippocampal neurons on micropatterns, it was shown that polarization does not occur when all neurites are constrained below 20 μm and that the longest neurite becomes the axon when only one neurite is allowed to grow freely [20,21].

The elongation timing model proposes that axonal differentiation depends on which neurite elongates first. This model is indirectly supported by the observation that the earliest extensively growing neurite usually becomes the axon. However, several independent studies in immature neurons at developmental stage 3 in vitro suggest that neurite elongation order does not strictly determine axonal fate. For example, isolated cases have been reported where an initially elongated neurite retracted and another subsequently elongated neurite became the axon [1]. In addition, experiments show that when a nascent axon is severed at its base, a different neurite can assume axonal identity [22], and that a second axon can be induced by towing, even when an axon is already present [23]. While intriguing, these findings do not definitively refute the elongation timing model, as they may represent rare events or experimental artifacts.

However, conventional culture systems have lacked the ability to independently manipulate neurite length and growth sequence in real-time, leaving this issue incompletely resolved. To address this limitation, we have developed a constructive neuroengineering platform based on modifiable agarose gel microfabrication technology [24,25,26,27,28]. This method enables stepwise and dynamic control of neurite elongation by precisely shaping and modifying thin agarose confinement layers during cultivation. Using this system, we can define neurite outgrowth directionality, length, and timing without applying external forces.

In this study, we applied this technology to systematically investigate the conditions for axon polarization as a function of neurite length and growth sequence in isolated rat hippocampal neurons. Specifically, we quantitatively determined the critical length thresholds for axon specification when only a single neurite was permitted to elongate and tested how the presence of a competing neurite alters these thresholds. Furthermore, we performed sequential elongation experiments to directly examine whether axon polarization depends on the elongation sequence or rather on the absolute and relative lengths achieved during cultivation.

## 2. Results and Discussion

### 2.1. Constructive Approach for Stepwise Elongation of Neurites in Agarose Gel Microstructures

To resolve whether axonal differentiation depends primarily on neurite length or elongation timing, we developed a constructive neuroengineering platform that enables precise, stepwise control of neurite number, length, and elongation sequence during cultivation. This platform utilizes a thin, spin-coated layer of low-melting-point agarose gel applied over a poly-D-lysine (PDL)-coated culture dish, providing a non-adhesive surface that can be selectively modified by photothermal etching.

Micropatterns are fabricated in situ by locally melting the agarose using a focused 1480-nm infrared laser integrated into a phase-contrast microscope system equipped with a motorized x-y stage. This process allows the exposure of adhesive PDL regions in precisely defined geometries, such as microchambers for soma placement and narrow microchannels to constrain neurite outgrowth.

The key feature of this approach is its ability to dynamically introduce new microchannels during cultivation, enabling stepwise and independent manipulation of neurite elongation paths and timing without applying external mechanical forces. Neurons adhere selectively within exposed PDL regions while neurite growth is confined by the surrounding agarose matrix, ensuring isolated and directional outgrowth.

**Figure 1:**
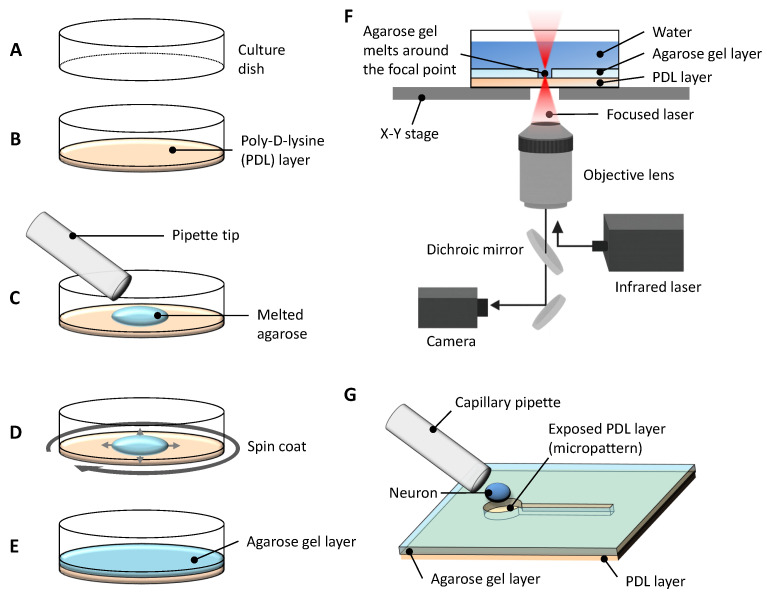
Schematic overview of the experimental system for agarose-based micropatterning and single-neuron placement. (**A**) A tissue culture dish treated with ion bombardment to enhance surface hydrophilicity was prepared for the experiment. (**B**) The bottom of the dish was coated with poly-D-lysine (PDL) to promote neuronal adhesion. (**C**) A drop of melted low-melting-point agarose was applied on top of the PDL coating. (**D**) The dish was spin-coated immediately to ensure uniform thickness of the agarose layer. (**E**) After solidification, the agarose gel layer provided a non-adhesive surface except at exposed PDL regions. (**F**) Micropatterns were fabricated via photothermal microfabrication: a focused 1480 nm infrared laser locally melted the agarose, exposing the underlying PDL in desired patterns while the dish was precisely moved on a computer-controlled x-y stage. (**G**) A single hippocampal neuron was placed in a defined microchamber using a fine capillary pipette for subsequent neurite outgrowth experiments.

This system allowed us to examine axon polarization under controlled conditions by independently defining the lengths and temporal sequence of neurite elongation during culture (Figure 1).

### 2.2. Critical Length for Axonal Differentiation in Single Neurite Elongation

To quantitatively define the neurite length threshold for axonal differentiation, we established a single-neurite elongation model under precisely controlled conditions. This setup enabled the elimination of competitive effects from multiple neurites and allowed rigorous evaluation of the relationship between neurite length and axon specification.

**Figure 2:**
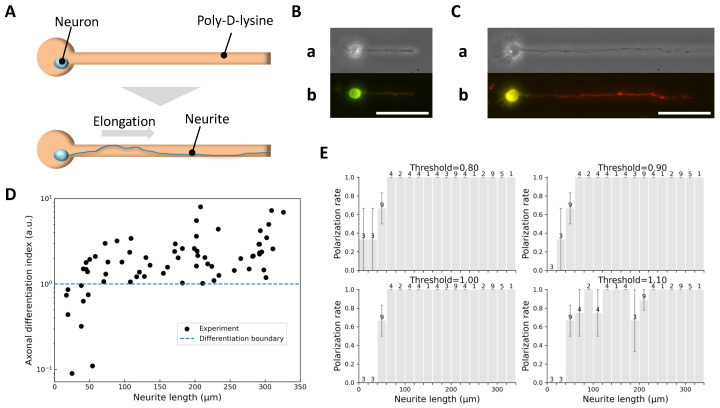
Identification of axons based on neurite length and immunofluorescence marker expression. (**A**) Schematic diagram of the single neurite elongation experiment: a micropattern with a single microchamber and microchannel was used to confine the soma and guide a single neurite along a defined path. (**B**) Example of short neurite growth. (**a**) Phase-contrast image showing a neurite extended to 48 μm on day 4 in vitro. (**b**) Corresponding immunofluorescence image with mixed expression of Tau-1 (red) and MAP2 (green), indicating undetermined identity. (**C**) Example of long neurite growth. (**a**) Phase-contrast image showing a neurite extended to 171 μm on day 4 in vitro. (**b**) Immunofluorescence image with strong Tau-1 and weak MAP2 expression, confirming axonal identity. Scale bars, 50 μm. (**D**) Axonal differentiation index ($I_{\mathrm{diff}}$) as a function of neurite length (68 neurites, obtained from 9 independent culture dishes). Neurites ≥ 58 μm consistently exhibited $I_{\mathrm{diff}}>1.0$, while neurites ≤ 38 μm showed $I_{\mathrm{diff}}<1.0$, supporting $I_{\mathrm{diff}}=1.0$ as a robust threshold for axon identification. (**E**) Histograms of polarization rate versus neurite length under different $I_{\mathrm{diff}}$ thresholds. The number of neurites within each length bin is shown above the corresponding bar. The four panels correspond to thresholds of 0.80 (**top left**), 0.90 (**top right**), 1.00 (**bottom left**), and 1.10 (**bottom right**). Thresholds of 0.90 and 1.00 yielded monotonically increasing trends, validating the choice of $I_{\mathrm{diff}}=1.0$ as a conservative and reliable criterion.

As illustrated in Figure 1, PDL-coated culture dishes were overlaid with a thin agarose layer and selectively patterned via infrared laser melting to form confinement structures comprising 40-μm microchambers and 10-μm microchannels. Neurons adhered only to PDL regions while agarose gel prevented adhesion, allowing selective confinement and guided outgrowth of individual neurites (Figure 2A).

Upon reaching target lengths, neurons were fixed and immunostained for MAP2 (green; somatodendritic marker, Figure 2B) and Tau-1 (red; axonal marker, Figure 2C), enabling quantitative classification via a defined axonal differentiation index $I_{\mathrm{diff}}$:

(1) $$I_{\mathrm{diff}}:=\frac{\left(I_{\mathrm{Tau-1}\max}+1\right)/\left(I_{\mathrm{Tau-1}\min}+1\right)}{\left(I_{\mathrm{MAP}2\max}+1\right)/\left(I_{\mathrm{MAP}2\min}+1\right)},$$

where maxima and minima represent the peak and background-subtracted fluorescence intensities along neurites. The offset of 1 prevents division by zero.

Figure 2D shows $I_{\mathrm{diff}}$ as a function of neurite length. Neurites ≥58 μm consistently exhibited $I_{\mathrm{diff}}>1.0$ and were classified as axons, whereas neurites ≤38 μm had $I_{\mathrm{diff}}<1.0$ and were considered undifferentiated. This analysis supported $I_{\mathrm{diff}}=1.0$ as a reliable threshold for axon identification. Additional histogram analysis (Figure 2E) confirmed that this threshold yielded a monotonically increasing polarization trend, whereas other thresholds (0.80 or 1.10) did not.

To further quantify the relationship between neurite length and the probability of axonal differentiation, we modeled the probability P(L) as a sigmoid function of neurite length *L*:

(2) $$P\left(L\right)=\frac{1}{2}\left(1+\tanh\left(\frac{L-L_{c}}{\sigma }\right)\right),$$

where $L_{c}$ is the critical length (corresponding to 50% differentiation probability) and σ characterizes the dispersion.

**Figure 3:**
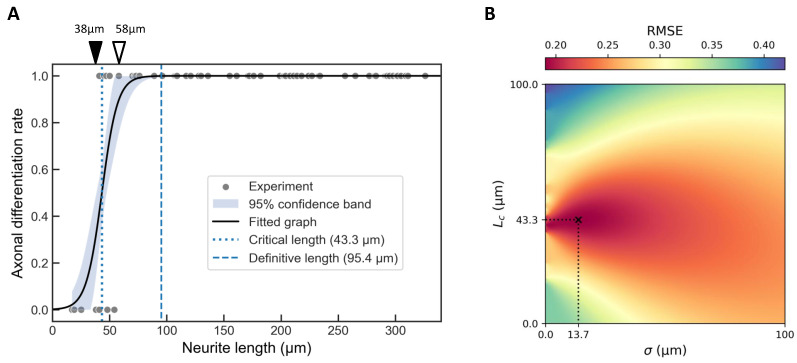
Critical length analysis for single neurite elongation. (**A**) Binary classification of neurites (axon = 1, non-axon = 0) based on data from Figure 2D, overlaid with a fitted hyperbolic tangent function (Equation (2)). The best-fitting parameters were $L_{c}=43.3$ μm and σ=13.7 μm. The shaded area represents 95% confidence band. The critical length $L_{c}$ (dotted line) corresponds to a 50% probability of axon specification, while the definitive length $L_{t}=95.4$ μm (dashed line) represents near-certain (100%) axon specification. Neurites measuring 38 μm or less (filled arrowhead) were not classified as axons, whereas those measuring 58 μm or more (open arrowhead) were consistently identified as axons. (**B**) Heatmap showing the root mean square error (RMSE) landscape for the hyperbolic tangent fit across the $L_{c}$ – σ parameter space. The global minimum, corresponding to the optimal fit with $R^{2}$ = 0.69, is indicated by a cross.

Fitting this model to the binary classification data (Figure 3A) yielded $L_{c}=43.3$ μm and σ=13.7 μm (Figure 3B). We additionally defined a “definitive length” $L_{t}=95.4$ μm, beyond which the probability of axon specification effectively reached 100%.

These results establish that neurite length alone serves as a key determinant of axonal fate when neurite elongation occurs in isolation, providing quantitative thresholds for future experimental and theoretical investigations.

### 2.3. Effect of Competing Neurites on Axonal Differentiation Thresholds

We next examined the simultaneous elongation of two neurites to test whether the critical length $L_{c}$ and definitive length $L_{t}$, established in the single-neurite condition, remain valid when competing processes are present.

**Figure 4:**
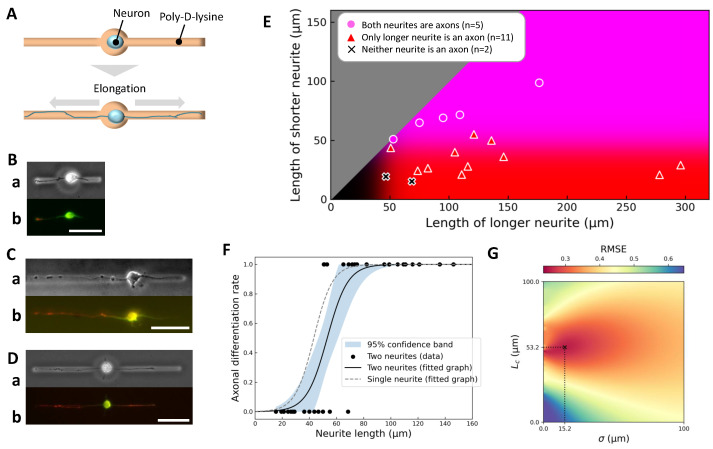
Axonal differentiation in simultaneous two-neurite elongation. (**A**) Schematic diagram of the simultaneous two-neurite elongation experiment. The soma was confined within a central chamber connected to two microchannels, allowing two neurites to elongate simultaneously. (**B**–**D**) Representative examples. For each, (**a**) shows a phase-contrast image just before immunofluorescence staining and (**b**) shows the final immunofluorescence image (green: MAP2, red: Tau-1). (**B**) Short–short pair (left: 47 μm, right: 19 μm); neither neurite differentiated into an axon. (**C**) Long–short pair (left: 121 μm, right: 55 μm); only the longer neurite became an axon. (**D**) Long–long pair (left: 109 μm, right: 72 μm); both neurites differentiated into axons. Scale bars, 50 μm. (**E**) Summary scatter plot (18 neurite pairs, obtained from 8 independent culture dishes), plotting the longer neurite length on the x-axis and the shorter neurite length on the y-axis. Background coloration reflects axon specification probabilities based on single-neurite elongation data: red intensity indicates likelihood of axon specification for the longer neurite, blue for the shorter neurite, and magenta (red + blue) for both neurites. (**F**) Length dependence of axonal differentiation probability in the two-neurite case compared to the single-neurite case. Black dots represent individual neurites; the black solid curve shows a hyperbolic tangent fit with best-fit parameters $L_{c}=53.2$ μm and σ=15.2 μm; the shaded area represents a 95% confidence band. The grey dashed curve shows the single-neurite fit, indicating a rightward shift of approximately 10 μm in the critical length due to competition between neurites. (**G**) Heatmap of the root mean square error (RMSE) for the tanh fit across the $L_{c}$ – σ parameter space. The global minimum, corresponding to the best-fit parameters with $R^{2}=0.73$, is indicated by a cross.

The experimental setup involved confining the soma within a central chamber connected to two opposing microchannels, allowing both neurites to extend simultaneously under identical conditions (Figure 4A). Example outcomes illustrate diverse behaviors depending on neurite lengths (Figure 4B–D). Specifically, when both neurites were short, neither differentiated into an axon (Figure 4B); when one neurite was long and the other short, only the longer neurite became an axon (Figure 4C); and when both neurites were long, both exhibited axonal marker expression (Figure 4D).

Summary data are presented as a scatter plot of neurite lengths (Figure 4E), overlaid with axon specification probabilities derived from the single-neurite condition. Consistent with previous results, neurites longer than the definitive length $L_{t}=95.4$ μm consistently became axons, regardless of the presence of a competing process.

However, quantitative analysis revealed that the fitted critical length $L_{c}$ increased from 43.3 μm (single-neurite case) to 53.2 μm when a second neurite was present (Figure 4F, black curve), suggesting that competition modestly elevates the threshold for axon specification. Furthermore, when the competing neurite itself exceeded $L_{t}$, the critical length further increased to 62.0 μm, indicating an additive competitive effect.

These findings suggest that inhibitory crosstalk occurs between neurites during simultaneous outgrowth, modulating the length-dependent axon specification threshold. This crosstalk may reflect resource competition, local signaling interactions, or feedback mechanisms that balance growth among processes originating from the same soma.

A heatmap analysis of the root mean square error (RMSE) for the fit (Figure 4G) confirmed the robustness of the estimated parameters, highlighting the reproducibility of this competitive effect.

Overall, these results demonstrate that while length remains the primary determinant of axonal differentiation, the presence of a competing neurite shifts the effective threshold upward, underscoring the context-dependent nature of axon specification.

### 2.4. Stepwise Control of Elongation Order and Its Effect on Axonal Fate

**Figure 5:**
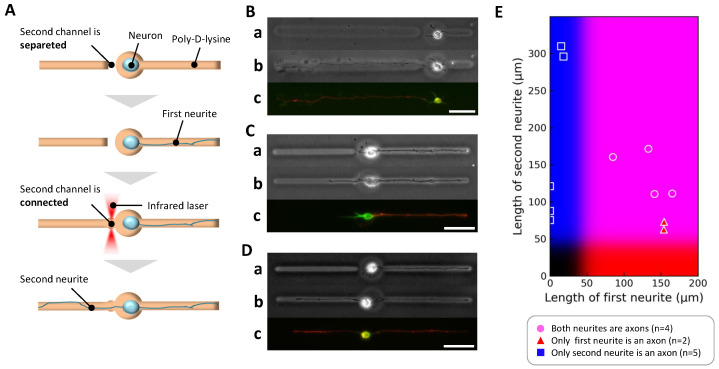
Axonal differentiation in stepwise two-neurite elongation. (**A**) Schematic diagram of the stepwise two-neurite elongation experiment. The soma was confined in a central chamber with one microchannel initially open, allowing a first neurite to elongate. After sufficient growth, the agarose barrier on the opposite side was removed, enabling a second neurite to extend. This design allowed investigation of axon specification under sequential rather than simultaneous neurite outgrowth conditions. (**B**–**D**) Representative examples. For each, (**a**) shows a phase-contrast image immediately before secondary microfabrication; (**b**) shows a phase-contrast image before immunofluorescence staining; (**c**) shows the final immunofluorescence image (green: MAP2, red: Tau-1). (**B**) Case where the first neurite grew to 58.0 μm by day 1, followed by second neurite extension to 316.0 μm by day 3; the initially growing neurite retracted to 16.5 μm, and only the longer second neurite differentiated into an axon. (**C**) Case where the first neurite extended to 154.8 μm by day 3, followed by second neurite extension to 63.7 μm by day 4; only the first neurite exceeded the threshold and differentiated into an axon. (**D**) Case where both neurites exceeded the threshold length (first: 165.0 μm by day 2; second: 111.4 μm by day 3), and both were classified as axons. Scale bars, 50 μm. (**E**) Summary scatter plot (11 neurite pairs, obtained from 7 independent culture dishes), plotting first neurite length (x-axis) versus second neurite length (y-axis). Background coloration reflects axon specification probabilities derived from the single-neurite condition: red intensity indicates likelihood of the first neurite being specified as an axon, blue for the second neurite.

To investigate whether the order of neurite elongation affects axon specification independently of neurite length, we implemented a stepwise paradigm uniquely enabled by our agarose-based microfabrication system. In this experiment, a neuron’s soma was initially confined within a microchamber connected to only one open microchannel, allowing a single neurite to elongate without competition. After this first neurite reached a target length, we removed the agarose barrier on the opposite side using photothermal etching to open a second microchannel, allowing a second neurite to subsequently elongate (Figure 5A). This design enabled precise decoupling of elongation order from neurite length—a capability not achievable with traditional culture methods.

Among eleven neurons analyzed, three distinct patterns of axonal fate emerged, exemplified in Figure 5B–D. In the first pattern, when the initial neurite was relatively short (<85 μm), the second neurite elongated further after its channel was opened and became the axon (Figure 5B). In the second pattern, when the initial neurite grew longer than 85 μm before the second neurite could elongate, the first neurite maintained its lead and became the axon (Figure 5C). In the third pattern, when both neurites eventually exceeded 85 μm, both exhibited axonal marker expression and were classified as axons (Figure 5D). These observations are summarized in the scatter plot of neurite lengths (Figure 5E), which shows that axon specification was consistently associated with the neurite that ultimately achieved the greater length.

These findings clearly indicate that neurite length, not elongation order, determines axonal fate even under sequential outgrowth conditions. The initially favored neurite could lose axonal identity if overtaken in length by a later-initiating neurite, underscoring the plasticity of axon specification processes. Importantly, this confirms that the cellular machinery for axon specification continuously assesses relative neurite lengths during early development rather than fixing axonal identity based solely on the temporal sequence of outgrowth.

This experiment exemplifies the power of our constructive neuroengineering platform, which enables dynamic, in situ modification of the cellular environment to test hypotheses that cannot be addressed in conventional static culture systems. The results reinforce the central conclusion that axon polarization is governed by relative neurite length at the time of decision, regardless of elongation history, highlighting the dynamic and context-dependent nature of early neuronal polarization mechanisms.

### 2.5. Advantages, Limitations, and Mechanistic Implications

This study establishes a novel constructive neuroengineering method enabling dynamic, stepwise control of neurite elongation sequences and lengths during cultivation, leveraging modifiable agarose gel microstructures. Using this approach, we quantitatively defined critical thresholds for axonal differentiation: a critical length ($L_{c}=43.3$ μm) corresponding to a 50% probability of axon specification, and a definitive length ($L_{t}=95.4$ μm) beyond which axonal fate was reliably determined. These thresholds align with prior indirect estimates [29] and approximate the axon hillock’s characteristic size [30].

In two-neurite elongation experiments, we confirmed that neurites exceeding $L_{t}$ consistently became axons; however, the presence of competing neurites shifted the effective critical length $L_{c}$ upward, suggesting inter-neurite inhibitory interactions that modulate axon specification thresholds. Notably, when both neurites surpassed $L_{t}$, dual axon formation was observed, indicating that competition does not preclude multiple axon identities under certain length conditions.

Stepwise elongation experiments provided direct evidence that axon specification depends on the relative and absolute neurite length rather than the order of elongation onset. This emphasizes neurite length as the dominant determinant of axon polarization, even when growth occurs sequentially, highlighting the plasticity and ongoing nature of the polarization decision-making process.

The primary advantage of our approach lies in its ability to dynamically and reversibly manipulate microenvironmental constraints during cultivation with high spatial and temporal resolution. Unlike conventional static micropatterning techniques, our modifiable agarose gel platform enables real-time, in situ reconfiguration of confinement structures, allowing systematic dissection of how neurite length and timing influence polarization outcomes. This flexibility will be critical for constructing designed neuronal circuits with prescribed geometries and polarity.

Our results support models in which axon specification emerges from length-dependent accumulation of molecular cues, such as Shootin1 gradients [31], and possibly mechanical or transport-based feedback mechanisms that reinforce polarization once a length threshold is crossed. The observation that thresholds shift in the presence of competing neurites suggests that axon specification is sensitive to the global context of neurite growth, consistent with dynamic resource allocation or molecular competition models.

The proposed model that neurite length is the dominant factor in axon specification may not fully account for the robustness of neural circuit formation during early development in vivo. For example, in cortical pyramidal neurons, axon specification has been proposed to follow the “Touch & Go” model [13,32,33]. According to this model, when a neurite of a multipolar cell makes contact with the pioneering axons of early-born neurons, it undergoes rapid elongation and differentiates into an axon. In such cases, physical contact with an existing axon may be sufficient to trigger axonal differentiation, without the neurite needing to reach a particular length threshold. Our constructive neuroengineering system could be extended to incorporate such factors by introducing controlled process–process contact, co-culturing with other neuronal or glial cell types. These adaptations would allow systematic testing of how the quantitative length thresholds identified here shift in more complex, physiologically relevant environments. Ultimately, this approach could provide a powerful platform for modeling neurological disorders associated with polarity defects [34].

Our axon identification relied on immunocytochemical markers (Tau-1/MAP2 ratios), and no electrophysiological confirmation was performed. This choice was unavoidable because we used isolated single-neuron cultures to eliminate confounding influences from other cells, enabling precise quantification of how neurite length and elongation order determine axonal differentiation. However, this isolation also means that the neurons have no synaptic partners, preventing the generation or propagation of synaptic activity that could be measured electrophysiologically. While patch-clamp recordings from axons are possible [35] and can confirm axonal identity by detecting active signal conduction, such measurements are technically challenging in our context. They are typically performed in tissue preparations or dense neuronal cultures, where neurons are embedded in a more physiologically supportive environment—receiving trophic support, extracellular signaling, and glial interactions that help sustain the high level of viability required for successful recordings. In our setup, neurons lack these supportive interactions, making them more physiologically vulnerable and less able to withstand the demands of axonal patch-clamp experiments. For these reasons, we have relied on Tau-1/MAP2 immunostaining as a robust and widely accepted morphological indicator of axonal identity. Future studies will aim to incorporate live-cell axonal markers and adapt the system for electrophysiological assessment to provide complementary functional validation.

By providing the first direct experimental demonstration that axon polarization can be predicted and manipulated based on neurite length under precisely defined conditions, this study lays critical groundwork for future efforts in constructive neuroengineering. Specifically, our findings advance toward the goal of achieving complete control over both axon and dendrite orientation and polarization in engineered neuronal networks—a key requirement for bottom-up construction of functional neuronal circuits with defined connectivity. The modifiable agarose gel platform developed here, thus, represents a powerful enabling technology for assembling neural circuits with predetermined architecture, polarity, and directional signal propagation.

## 3. Conclusions

In summary, we developed a constructive neuroengineering approach using modifiable agarose gel platforms that enables precise, dynamic control over neurite elongation length and timing in cultured neurons. By systematically manipulating and quantifying neurite growth under controlled conditions, we demonstrated that axon polarization is governed primarily by neurite length rather than elongation order, even in the presence of competing neurites. This work provides the first direct experimental evidence establishing quantitative length thresholds for axonal differentiation and reveals context-dependent modulation by inter-neurite interactions. Our findings lay the groundwork for achieving complete directional control over axons and dendrites, a key step toward bottom-up assembly of functional neuronal circuits using engineered substrates.

## 4. Materials and Methods

This study was conducted in accordance with the Act on Welfare and Management of Animals of the Ministry of the Environment, Japan. All animal experiments and protocols were approved by the Waseda University Animal Experiment Committee (permission numbers: A23-096 and A24-099) and adhere to ARRIVE 2.0 guidelines.

### 4.1. Culture Dish Preparation

Agarose gel microstructures on the 35 mm tissue dish (300-035, AGC Technoglass Co., Ltd., Shizuoka, Japan) were prepared as follows. First, the 35 mm dish was made hydrophilic with a plasma ion bombarder (PIB-20, Vacuum Device Inc., Ibaraki, Japan). Each dish bottom was then covered with 100 μL of 1 mg/mL poly-D-lysine (P0899, Sigma-Aldrich Co., LLC., Tokyo, Japan) for 15 min to enhance cell adhesion. After incubation, the dishes were rinsed three times with sterile water to remove excess PDL and air-dried for 15 min.

Next, 1 mL sterilized water was added to each dish, and 650 μL of the water was removed to leave a thin residual layer. Then, 85 μL of 3.5% low-melting point agarose gel (melting point = 65 °C; E-3126-25, BM-BIO BM Equipment Co., Ltd., Tokyo, Japan) was applied to the dish surface and evely spread using a spin coater (1H-D7, MIKASA, Tokyo, Japan). The spin coating protocol included an initial spin at 500 rpm for 3 s followed by 3000 rpm for 18 s. After coating, 2 mL of water were added to keep the gel hydrated, and the dishes were cooled to solidify the agarose layer.

### 4.2. Photo-Thermal Microfabrication of Agarose Gel

Photothermal microfabrication was performed with a specialized 1480 nm infrared laser etching system. The setup combined three main components: a phase-contrast microscope (IX-71 with 20× phase-contrast objective lens, LCUPlanFL N, OLYMPUS, Tokyo, Japan), a motorized x-y stage (BIOS-206T, SIGMA KOKI Co., Ltd., Tokyo, Japan), and a 1480-nm focused laser irradiation module (RLM-1-1480, IPG Laser, Oxford, MA, USA). During microfabrication, two wavelengths were used simultaneously: 520 nm visible light for phase-contrast imaging and 1480 nm infrared light for agarose etching. Phase-contrast images were captured with a CCD camera (CS230, Olympus). Dichroic mirrors and optical components were selected to ensure efficient transmission and reflection at both wavelengths.

To produce micropatterns, the agarose surface of each dish prepared as mentioned above was irradiated with 1480 nm focused laser, locally converting the gel into a sol state. The desired patterns were created by moving the culture dish along the x-y plane with the computer-controlled motorized stage, allowing accurate and reproducible microstructure fabrication. The heating effect on the underlying PDL coating was minimal due to the low melting point of the agarose, allowing cells to adhere reliably to the exposed PDL surface after patterning.

### 4.3. Cell Cultivation

Primary rat hippocampal neurons were isolated and purified from embryonic day 18 Wistar rat embryos (Tokyo Laboratory Animal Science, Tokyo, Japan). Tissue dissociation was performed using neuron dissociation solutions (291-78001, FUJIFILM Wako Pure Chemical Co., Osaka, Japan), with enzymatic digestion followed by density gradient centrifugation. Individual neurons were placed with a fire-polished fine glass pipette onto each microchamber of a dish prepared as mentioned previously. All glass pipettes were pre-washed by the manufacturer using an ultrasonic treatment to minimize contamination.

Cells were cultured with neuron culture medium (148-09671, FUJIFILM Wako Pure Chemical Co., Osaka, Japan), and cultures were maintained at 37 °C under 5 % CO_2_ at saturated humidity. The Neurobasal-based culture medium used in this study relies on sodium bicarbonate buffering, making CO_2_ supplementation essential. Hence, for laser microfabrication performed after neuronal seeding, each dish was processed within 5 min at room temperature and promptly returned to the incubator.

Cells were maintained until they extended neurites or pairs of neurites reaching target lengths. Consequently, the culture duration varied between 1 and 7 days depending on the experimental condition. It should be noted that the impact of cultivation time itself was beyond the scope and limitation of this study.

### 4.4. Cell Observation

Neurons on the pattern were observed using inverted optical microscopy (IX-71 with an 20× phase-contrast objective lens, LCPlanFl, OLYMPUS, Tokyo, Japan) equipped with a CCD camera (1501M-GE, THORLABS, Newton, NJ, USA). Images were acquired regularly to monitor neurite growth.

### 4.5. Immunofluorescence Staining

The immunostaining was conducted using a modified version of protocols described in our previous studies [26,27]. For axon identification, primary staining was performed with Anti-Tau-1 antibody (MAB3420, Sigma-Aldrich Co. LLC., Tokyo, Japan), followed by a Goat anti-Mouse IgG2a Alexa Fluor 555 secondary antibody (Thermo Fisher Scientific, Waltham, MA, USA). For dendrite labeling, primary staining was performed with Anti-MAP2 antibody (SIGMA ALDRICH M4403, mouse monoclonal IgG1), followed by a Rabbit anti-Goat IgG Alexa Fluor 488 (Thermo Fisher Scientific) as the secondary antibody. Fluorescence images were recorded with the same CCD camera imaging system used for phase-contrast observation.

### 4.6. Quantification and Data Analysis

Phase-contrast images in TIFF format were analyzed using ImageJ 1.54 [36] to measure neurite lengths. Although the microchambers were generally effective in restricting neurite outgrowth, samples in which neurites extended to more than twice the soma diameter were excluded from the analysis.

Fluorescence images were also analyzed with ImageJ to calculate the axonal differentiation index $I_{\mathrm{diff}}$, which is defined in the Equation (1). Since all neurites longer than 58 μm had the value greater than 1.0, this threshold was adopted to classify axonal differentiation. As a result, the data yielded a scatter plot of axonal differentiation vs. length. The resulting data were plotted as scatterplots showing axonal differentiation versus neurite length. Curve fitting to a hyperbolic tangent function was performed using the optimize.curve_fit function from the SciPy 1.15.3. [37] library on Python 3.12, and additional analyses and visualizations were carried out with NumPy [38], pandas [39], matplotlib [40], and seaborn [41].

## Data Availability

Jupyter notbooks used to analyze the data are available at https://github.com/soyahagiwara/Constructive-Neuroengineering-of-Axon-Polarization-Control.git accessed on 15 August 2025.
